# The influences of nursing transformational leadership style on the quality of nurses’ working lives in Taiwan: a cross-sectional quantitative study

**DOI:** 10.1186/s12912-015-0082-x

**Published:** 2015-05-14

**Authors:** Ping-Yi Lin, Sara MacLennan, Nigel Hunt, Tom Cox

**Affiliations:** 1 Division of Psychiatry and Applied Psychology, School of Medicine, University of Nottingham, Jubilee Crescent, Wollaton Road, NG8 1BB Nottingham, UK; 2 Transplant Medicine & Surgery Research Centre, Changhua Christian Hospital, No.135, Nansiao St., Changhua city, Changhua county 50006 Taiwan; 3 Academic Urology Unit, University of Aberdeen, oresterhill, Aberdeen, AB25 2ZD Scotland UK; 4 Centre for Sustainable Working Life, Birkbeck, University of London, Malet Street, Bloomsbury, London, England WC1E 7HX United Kingdom

**Keywords:** Transformational leadership, Job satisfaction, Organisational commitment, Quality of working life

## Abstract

**Background:**

Taiwan’s NHI system is one of the most successful health care models for countries around the globe. However, little research has demonstrated the mental health issues associated with nursing transformational leadership style under the NHI system, especially in the quality of nurses’ working lives in Taiwan. It is important to know the relationship between transformational leadership style and the mental health of nurses, organisational commitment and job satisfaction. The research aimed to understand the influences of nursing transformational leadership style on the quality of nurses’ working lives in Taiwan. The research hypothesis was that transformational leadership styles would have positive influence on the quality of nurses’ working lives.

**Methods:**

This was a cross-sectional quantitative study. Nurses from each type of hospital ownership (private, public and religious) were recruited. Participation was voluntary and signed informed consent was obtained. The inclusion criteria were nurses with at least one year’s work experience in the hospitals. Self-administrated questionnaires were used. A total of 807 participants were contacted and 651 questionnaires were fully completed (response rate 80.7 %). A theory driven model was used to test the research hypotheses using structural equation modelling performed with AMOS 16.0.

**Results:**

Transformational leadership contributes significantly to supervisor support. Workplace support, particularly from the supervisor, is an important mediator variable that explains the relationship between transformational leadership and job satisfaction. Organisational commitment was the strongest factor relevant to the general health well-being in Taiwanese nurses than job satisfaction. The hypothesized positive relationships between transformational leadership and all variables were supported by the data.

**Conclusions:**

Our findings have important consequences for organisational health. Our model demonstrates a complete picture of the work relationships on the quality of nurses’ working lives. The results provided information about the subordinates’ perceptions of transformational nursing leadership styles and mental health outcomes in different hospital settings, as well as identified organisational factors that could improve the quality of nurses’ working lives.

## Background

Nursing is facing an increasing shortage of employees worldwide and Taiwan is no exception. [1, 2]. Numerous studies have demonstrated that nursing is a stressful occupation [3–5]. Organisational factors such as leadership style, social support, and work climate are contributing factors to well-being of nurses [6–8]. Regarding ownership, hospitals in Taiwan can be briefly classified into three types. These types of hospitals might be similar in their environment in terms of location, the structure of the building, the number of beds, facilities and organisational structure. The management system and environmental climate might be different in these hospitals due to the ownership. The hospital management system is unlikely to be the same in the different types of hospital. Although, how management systems impact on their employees, in terms of their job performance and the quality of their working lives, needs consideration. Recently, a growing body of research which demonstrates that subordinates’ perceptions of their working conditions are related to leadership behaviour [9].

Nursing is often defined as the kind of job with long working hours and high job stress [10]. Such factors as low job control, high job demands and low supportive work relationships are correlated with stress in nurses [11] and high rate of staff turnover [12]. Research has showed that nursing transformational leadership style is associated with employees’ job satisfaction [13] and general health well-being [14], though the mechanism between nursing transformational leadership style and the quality of nurses’ working lives is not clear. There is a need to understand the mechanism between transformational leadership style and quality of working life in health care professions. Therefore, we propose a theory-driven model to examine this mechanism by using structural equation modelling.

### Transformational leadership style and supervisor support

Transformational leaders stimulate their subordinates to share a vision and use goals as inspirational motivation. Subordinates are encouraged to think of old problems in new ways and are seen individually. Additionally, followers are influenced by a transformational leader who is trustworthy, respectable and dedicated. By applying transformational leadership, leaders can confidently deal with a complex and rapidly changing working environment. The effectiveness of transformational leadership is evident.

**Fig. 1:**
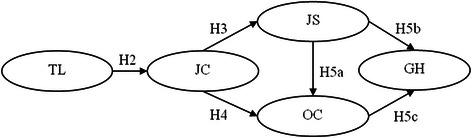
The proprosed path model of relationships. Notes: TL = Transformational Leadership Style; JC = Job Content; JS = Job Satisfaction; OC = Organisational Commitment; GH = General Health Well-being

Supervisor support may be one of the significant factors in keeping nurses satisfied in their work [15]. Nursing supervision has a positive influence on nurses’ well-being and their ability to cope with their stressful work situations [16]. We build on previous research by examining whether the relationship between transformational leadership and the quality of nurses’ working lives was mediated by subordinates’ perceived support from their supervisors. We proposed a conceptual model which is comprised of five key variables. The model is presented in Fig. 1. We assumed that transformational leadership style will have either a negative/positive influence on the general health well-being of nurses and that the relationship will be mediated by support within the organisational system. In addition, the effects of transformational leadership style on the general health well-being of nurses will be buffered by job satisfaction and organisational commitment.

#### The role of leadership in support

Leaders should be able to influence and inspire others, develop strategies, organise resources and empower people [17]. The leader is in a position with multiple dimensions, such as being a clinical expert in their field as well as being a manager in a hospital. In the nursing setting, change management, negotiating ability and conflict management are essential skills that nurses should develop to become effective leaders [18]. There is no optimal leadership style, as the personality of the leader, subordinates, and the situation can all influence leadership effectiveness [19]. The perceptions of leaders and subordinates are also inconsistent, with subordinates often preferring leaders with more clearly expressed leadership behaviour than leaders themselves prefer to demonstrate [20]. Leadership style may affect the performance of nurses directly and may have positive or negative effects. It is also associated with staff turnover [21, 22]. Leadership/management style is one of the main sources of distress for nurses [23]. It is also suggested that occupational stress has an adverse influence on the quality of working life for nurses [24].

Taken together, leadership may affect both nurses’ work performance and their quality of the working life. Developing a transformational nursing leadership style is an alternative organisational strategy to improve nurse performance and promote better patient care outcomes [25]. It may further enhance the quality of the working life of nurses.

The following hypotheses examined the main effects of transformational leadership styles on nursing mental health outcomes:

*Hypothesis 1:* Transformational leadership styles are related to nursing mental health outcomes.

Transformational leadership style is associated with subordinates’ working conditions [26]. Training managers in transformational leadership style may have a positive influence on health care workers’ mental health over time [27], although it may present an incomplete picture of the impact of work and the relationship on well-being without considering working conditions [28]. Job content is also included as one of the variables in the proposed model.

*Hypothesis 2:* The higher the level of transformational leadership the higher the level of perceived supervisor support.

#### Follower work attitudes

Work attitudes have a strong influence on the quality of nurses’ working lives. Job satisfaction and organisational commitment are outcome variables of nurses’ work attitudes. Job satisfaction concerns how an employee feels about his or her job and is related to organisational commitment. These are important to the quality of an employee’s working life. Factors such as job content, job satisfaction and organisational commitment are presented in the proposed model. Job satisfaction is a predictor of the quality of nursing working life. In contrast, job dissatisfaction can be the main predictor of intention to leave nursing [29].

Leadership, particularly the transformational leadership style, is associated with higher levels of job satisfaction [30]. Transformational leaders may help ensure employees’ job satisfaction and psychological well-being [31, 32]. In Taiwan, nursing directors display transformational leadership more frequently in their workplaces and nursing faculty staff exposed to this leadership style are moderately satisfied in their jobs [33]. Nurses with supervisors employing a transformational leadership style were less likely to experience decreased job satisfaction than nurses with supervisors employing other styles [34]. Therefore, supervisors presenting a transformational leadership style can be perceived as providing supervisor support in the organisation. Job satisfaction and organisational commitment are two factors which moderate the effects of general health well-being and transformational leadership style. We aimed to test the relationship among supervisor support, job satisfaction, and organisational commitment.

*Hypothesis 3:* The higher the level of supervisor support will indicate a higher level of job satisfaction.

*Hypothesis 4:* There will be a positive correlation between supervisor support and organisational commitment.

Organisational commitment is highly associated with the quality of nurses’ working lives. One of the most important perspectives is to define a proper methodology for an overall assessment of the quality of the working life of nurses. The aim of the present study was to explore nursing leadership style and its relationship between mental health outcomes of nurses.

*Hypothesis 5a:* The higher the level of job satisfaction, the higher the level of organisational commitment.

*Hypothesis 5b:* The higher the level of job satisfaction, the lower the level of poor general health well-being.

*Hypothesis 5c:* The higher the level of organisational commitment, the lower the level of poor general health well-being.

According to Karasek’s theoretical model [35], job demands and work control are two important work environment elements. In this approach, high job strain is viewed as the consequence of an individual’s ability to cope with high job demands under conditions of low control. Payne and Fletcher [36] stated that less effort is needed to cope with high job strain if support is available in the organisational system. Karasek’s model was reconceptualized to include social support and was used widely to measure work characteristics and job strain. High levels of support from supervisors and co-workers play a moderating role against the negative effects of high strain jobs on levels of work performance [37], so supervisors will have a major influence on employees’ mental health. In addition, supervisor support might play a mediator role in raising the positive effects of the quality of the working life of nurses. Thus, it might be possible to improve organisational health by enhancing the leadership styles of supervisors.

As stated before, the general aim of the present study was to explore nursing leadership style and its relationship between the mental health outcomes of nurses. The results are presented in two parts: data analysis and model development. In the first part, a basic background of the quantitative phase of the study is described. The second part of the study describes the utility of structure equation modelling (SEM).

## Method

### Research design

This was a cross-sectional quantitative study. The data were collected using a self-report questionnaire that consisted of six sections: demographic information, leadership style, job content, general health well-being, organisation commitment and job satisfaction. Demographic data were obtained, which included age, gender, marital status, grade of nursing practice, educational level, working experience and work tenure.

### Sampling Strategy

The surveys were conducted in Chinese and all scales were pilot tested. The participants were selected from 12 hospitals in Taiwan, 4 hospitals in each type of ownership. Participation was voluntary and signed informed consent was obtained. The nurses’ eligibility to participate in this study were those graded from N1 (basic training) to N4 (specialized training and research) and those with at least one year’s work experience in their current hospital. The overall response rate was 80.7 %. A total of 651 valid questionnaires were completed and returned. Participant demographic characteristics are presented in Table 1. A total of 41.5 % respondents worked in private hospitals, 34.4 % worked in public hospitals and the remaining 24.1 % worked in hospitals run by religious organisations. The mean age of the participants were 30.5 *(SD = 6.2*) years. The average work tenure was 4.5 (*SD = 4.5*) years. The majority of respondents were female and more than half of them were single. In terms of grade of nursing practice and educational level, there was a statistically significant correlation among the three types of hospital.

**Table 1:** The mean scores of variables among three types of hospital ownership

	Private hospital	Public hospital	Religious hospital	*P*	Overall
	M, SD	M, SD	M, SD		M, SD
	(Range)	(Range)	(Range)		(Range)
Transformational Leadership	32.50, 10.76	32.98, 12.12	31.24, 11.43	NS	32.36, 11.41
	(0–52)	(0–52)	(0–52)		(0–52)
Supervisor Support	7.22, 2.18	7.27, 2.35	7.06, 2.31	NS	7.20, 2.27
	(0–12)	(0–12)	(0–12)		(0–12)
Job Satisfaction	45.38, 7.21	46.49, 7.54	46.12, 7.18	NS	45.94, 7.33
	(17–70)	(12–72)	(25–71)		(12–72)
Organisational Commitment	49.57, 11.93	54.33, 11.49	55.50, 11.77	<0.001	52.64, 12.01
	(8–76)	(14–83)	(21–84)		(8–84)
General Health Well-being	15.07, 5.00	14.52, 5.15	13.20, 5.09	0.001	14.43, 5.12
	(1–32)	(0–31)	(2–26)		(0–32)

### Measures

#### Nursing leadership style

Participants completed the Multifactor Leadership Questionnaire [38], which measures the following dimensions of transformational leadership: idealized influence, inspiration motivation, intellectual stimulation, and individualized consideration. Participants were asked to indicate their degree of perception about transformational leadership style regarding their leader (one level above) by using a four-point Likert scale ranging from ‘not at all (0)’ to ‘frequently, if not always (3)’. Cronbach’s alpha (α) for transformational leadership was .975 which demonstrates very good reliability (an alpha of greater than .70). In the present study, both composite reliability and the value of the average variance extracted (AVE) are employed to assess convergent validity. Adequate convergent validity is assessed with reliabilities above .80 and the value of AVE above .50 [39]. The value of the composite reliability in the transformational leadership scale is .98 (exceeding .80) and the value of AVE is .70 which can be seen as good convergent validity.

#### *Job content*

Job content was measured using Karasek’s Job Content Questionnaire (JCQ) [40]. This instrument comprised of 22 items and three dimensions: job control, personal demands, and workplace support. The job control scale is the sum of the two subscales, skill discretion and decision authority. The workplace support scale is the sum of the two subscales, supervisor support and co-worker support. Participants were asked to indicate social and psychological characteristics of their jobs using a four-point Likert scale ranging from strongly disagree (0) to strongly agree (3). Cronbach’s alpha was .721 for the Job Content Questionnaire. In the job content scale, the value of the composite reliability was .89 in the supervisor support scale and the value of AVE is .68 which is an acceptable level of convergent validity.

#### *Job satisfaction*

In the present study, the job satisfaction scale of the Occupational Stress Indicator (OSI; [41] was employed. It included 22 items in its original form. As it was conducted with nurses who only read and spoke Mandarin, the Chinese version of the job satisfaction scale which comprised 12 items from the OSI, was used. The Chinese version of job satisfaction has been tested and validated in a Taiwanese population [42]. Participants were asked to rate their degree of satisfaction about their jobs using a six-point Likert scale ranging from very unsatisfied (1) to very satisfied (6). Cronbach’s alpha was .939, the value of the composite reliability was .94 and the value of AVE was .57 which is an acceptable level of convergent validity.

#### *Organisational commitment*

Organisational commitment is the level of psychological and social attachment an individual has to an organisation. Organisational characteristics are key factors in nurse attraction and retention. One of the main approaches to measuring organisational commitment in health care professionals is the Organisational Commitment Questionnaire (OCQ), which is a 15 item questionnaire designed to describe global organisational commitment as a total commitment scale [43]. A seven point Likert scale ranging from strongly disagree (0) to strongly agree (6) was used to indicate degree of organisational commitment. Cronbach’s alpha was .878. A total scoring method was performed in this scale.

#### General health well-being

The 12-item General Health Questionnaire [44] was developed to measure non-specific psychiatric disorders. The GHQ-12 (Chinese version) has been tested and validated in Chinese societies [45]. In the present study, the GHQ-12 was applied to measure the perceptions of the participants regarding their health status. Responses were coded by using an un-weighted four-point Likert method (0-1-2-3). Cronbach’s alpha was .815 for the GHQ.

### Ethical considerations

Ethics approval was granted from the Institute of Work, Health and Organisations at the University of Nottingham and from the Institutional Review Board at Changhua Christian Hospital (IRB, Taiwan).

### Statistical analysis

Pearson correlations were used to detect the correlations of all the variables in the model. Analysis of variance (ANOVA) was used for continuous parametric data to detect the differences among the three types of hospital. Scheffe’s post hoc tests were applied for comparisons of means which involve more than two means at a time. SEM as used to examine the pattern of the relationships among all variables. Goodness-of-fit is evaluated by using those fit indicators as well as the comparative fit index (CFI), nonnormed fit index (NNFI/TLI), and the root mean square error of approximation (RMSEA) [46, 47]. To assess model fit, the applied fit indices are the values of chi-square where > .05 indicates a good fit [48]. Values of below .08 for RMSEA indicate acceptable fit [49]. In addition, values of CFI and NFI > .90 indicates a good fit [50]. SEM was applied in the assessment of each construct and respective items which included the measurement model. In the assessment of the measurement model, SEM has been proven to have more advantages than multiple regressions in evaluation the model fitting. SPSS version 16.0 was used to perform exploratory factor analysis (EFA) and AMOS (Analysis of Moment Structure) version 16.0 [51] was used to perform confirmatory factor analysis (CFA). EFA was used for model development and CFA was used for model testing. Hypothesis testing involved that conducting an evaluation of whether a SEM was consistent with the data or not. To following fit indices were used in this study such as Chi-square (*χ*^2^), RMSEA, CFI, TLI and NFI.

## Results

Regarding the internal reliability of the five subscales, Cronbach’s alpha (α) scores for each subscale exceeded the level of .70. In order to test Hypotheses 1 and 2, correlation analyses were conducted. The bivariate correlations are displayed in Table 2, which shows means, standard deviations, and the correlations of all the variables in the model. All correlations of the variables are statistically significant The strongest correlation was between supervisor support and transformational leadership style (*r* = .735). Thus, hypotheses 1 and 2 are supported.

**Table 2:** Correlations for all the variables in the proposed model

Variables	Correlations				
	1	2	3	4	5
1. Transformational leadership	1				
2. Supervisor support	.735**	1			
3. Job satisfaction	.475**	.518**	1		
4. Organisational commitment	.321**	.359**	.552**	1	
5. General health well-being	−.151**	−.169**	−.289**	−.389**	1

** Correlation is significant at the 0.01 level

Work attitude variables, job satisfaction and organisational commitment were significantly correlated with general health status. High scores of job satisfaction or organisational commitment are associated with better health status.

There was a statistically significant difference in both organisational commitment level and general health well-being between the three types of hospitals (see Table 1). An analysis of variance (ANOVA) showed that the scores of organisational commitment was significant, *F* (2,648) *=* 16.254, *p <* .001. Post hoc analyses using the Scheffé post hoc criterion for significance indicated that the average number of errors was significantly lower in the private hospitals (*M* = 49.57, *SD* = 11.93) than in the other two ownership hospitals (public and religious). Regarding the scores of general health status, it was significant statistically in three ownership hospitals, *F* (2,648) *=* 6.798, *p =* .001. The scores were significantly higher in private hospitals (*M* = 15.07, *SD* = 5.00) which revealed the worse health status. However, there was no statistically significant difference in job satisfaction among the three hospitals. These results represented the degree of organisational commitment with the highest scores found in the religious hospitals when compared with the other hospitals.

### Model development

#### Proposed model

SEM was used to test the proposed model fit among all the variables. Figure 1 presents the initial model of the present study. This model was used to test the relationship between transformational leadership styles of nursing and nursing health outcomes. This model consists of structural parameters for the following: 1) direct path between transformational leadership and supervisor support; 2) direct path between transformational leadership and job satisfaction; 3) indirect path between supervisor support, job satisfaction, and general health well-being; 4) indirect path between transformational leadership style and all variables. Fit of the overall model was assessed (Chi square = 0.241; df = 3; *p* = .971) and the fit indices (CFI = 1; TLI = 1.009; NFI = 1.000) have a value greater than 0.9 which indicates good model fit. In addition, the value for RMSEA is less than .05 (RMSEA = .000). This implies a good model fit. The model was tested by estimating all the hypothesised paths. Non-significant paths were dropped in a stepwise fashion so that only significant paths were presented. Although, these results showed a near-perfect model fit. According to the small degrees of freedom (df = 3), it might caused by all the variables are identified. However, from the results of a good model fit with large sample size, it strengthens the hypotheses of the conceptual model.

**Fig. 2:**
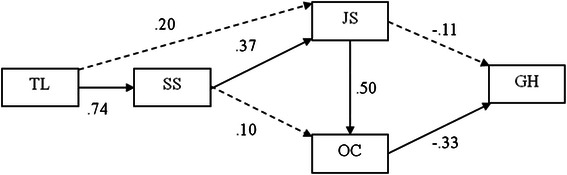
The final model of the study. Notes: TL = Transformational Leadership Style; SS = Supervisor Support; JS = Job Satisfaction; OC = Organisational Commitment; GH = General Health Well-being

The results presented in Fig. 2 show that transformational leadership had direct influence on supervisor support (β = .74).

More transformational leadership behaviours are related to greater supervisor support. Greater supervisor support is related to greater job satisfaction (β = .37), which in turn is positively related to greater organisational commitment (β = .50). Thus, hypotheses 3-5a are supported. According to these results, supervisor support has a mediation effect on the relationship between transformational leadership behaviours and job satisfaction. In addition, the effect of supervisor support on the level of organisational commitment was buffered by job satisfaction. Similarly, the results shown in Fig. 2 indicate that a higher level of job satisfaction was related to better general health well-being (β = −.11). Thus, Hypothesis 5b was supported. More commitment to the organisation had better general health well-being (β = −.33). Thus, hypothesis 5c was supported.

## Discussion

Transformational leadership continues to predominate in health care studies [52–54]. Nevertheless, a growing body of nursing research has addressed the significance of the relationship between transformational leadership and job satisfaction of nurses. Ideally, transformational leadership will create a motivating vision and enhance job performance. However, the relationship between transformational leadership and work related mental health variables are not clearly understood. In this study, we expected the transformational leadership styles of nursing to influence nursing mental health outcomes. Based on the main hypotheses of the research, the results were presented in the final model which revealed the positive relationship between nursing transformational leadership and general health status. The results also showed that supervisor support plays a mediating role between transformational leadership styles and job satisfaction. Supervisor support has a dramatic influence on employees’ job satisfaction compared with other factors. In comparison to work demands, supervisor support plays an important role; in that it is more likely to influence health outcomes of nurses in terms of job characteristics. Supervisor support was one of the job characteristics highly associated with transformational behaviour. Supervisor support is a core transformational behaviour and it increases job satisfaction of nurses. This result is concordant with previous studies [55]. The subordinates tend to have a higher level of job satisfaction when they perceive supervisor support as a specific behaviour of transformational leadership styles. This provides a valuable contribution to the subordinates’ perceptions of transformational leadership styles in terms of job satisfaction. Furthermore, leadership is an interactive process between the leader and the subordinate. Subordinates’ perceptions of a leader are important as they reveal information about how subordinates perceive the behaviours of the leader and the causal relations by which these perceptions translate into the mental health outcomes of the subordinate. In our study, transformational leadership style also plays an important role in both job satisfaction and organisational commitment of nurses. Regarding the quality of nursing working life, job satisfaction and organisational commitment are particularly influential.

Regarding demographic background, the age of the Taiwanese nursing workforce tended to be younger compared to those in Western countries [56]. The average age of employed nurses was 30.5 (*SD = 6.2*) years. However, in western developed countries, the average age of employed nurses was more than 40 years [57]. This reveals that the nursing profession is more likely to face difficulty with retaining its workforce in Taiwan. Hu et al.[58] reported that overtime is one of the predominant outcomes related to the nursing workforce. In addition, excessive workload has been reported by 44.8 % of Taiwanese nurses as one of the main reasons for quitting their jobs [59]. This may explain unfavourable working nature for retaining nurses. Regarding the ownership of the hospital, it is more likely that ownership had less influence on job satisfaction of nurses. Job satisfaction scores reported by the nurses were lower than other professions in Taiwan [60]. Regarding organisational commitment, the lowest scores on organisational commitment between the three types of hospital were found in private hospitals. Similarly, hospital ownership was associated with organisational commitment [61]. This implies that organisational culture/climate could be a potential factor influencing organisational commitment. There was a positive correlation between job satisfaction and organisational commitment, a similar result reported by Blegen [62]. Organisational commitment is more closely related to mental well-being than job satisfaction.

### Strengths and limitations

The present study produced a number of findings that illustrated the influences of transformational leadership in Taiwanese nurses and provided a comprehensive overview of work related mental health outcomes. The large sample size (*N* = 807) and the high response rate (80.7 %) indicates that the allowance of category variable detection and the effect of response bias were likely to be minimal. This study also contributes to the Taiwanese nursing field as well as a starting point for researchers from which they can further develop this model within the nursing profession.

Although transformational nursing leadership has been investigated in Western countries [63, 64], there have been many published studies that develop a hierarchal model using SEM to explore the relationships between transformational leadership, job content, work attitudes and general health status. However, the relationships among them still have not been clear defined.

There are limitations of the present study. There may be other moderating factors which influence the general health status of nurses, such as family demands, work condition and climate. In terms of job satisfaction and organisational commitment, several characteristics are significant in relation to job satisfaction (ethnicity, gender), organisational commitment (patient load, mandatory overtime, shifts, and unit type) and intent to stay on job (income, age). In addition, as in all cross-sectional studies, we only took measures once and relied on self-administered questionnaires which may lead to bias. However, with the substantial sample size and high response rate, the potential biases are minimized.

### Implications for future research

Further research based on the results of this study is essential. Training nursing leaders to apply transformational leadership behaviours in the workplace might have a positive impact on the mental health outcomes of nurses. Future research should design educational programmes as interventions which aim to teach how to use transformational leadership in a hospital setting. Although much research has focused on work stress in health care professionals such as nurses, most of the studies are focused on individual interventions such as stress management. Therefore, conducting transformational leadership education programmes as organisational interventions in future research may enhance employees’ satisfaction and commitment to their organisation.

An appropriate leadership style can lead to better emotional well-being of nurses and improved organisation commitment. Workplace mental health promotion programmes and legislations can act as a buffer to enhance mental health outcomes. The main implication of the findings is that they provide a deeper understanding about the relationship between nurse leadership and nurses’ mental health outcomes. On a more practical level, the findings could also be used as a reference to improve leadership styles and further promote organisational health.

## Conclusion

The aim of this study was to gain a better understanding of the relationship between transformational leadership and work related mental health variables of nurses in Taiwanese hospitals. This study focused on revealing the relationships between the key variables including transformational leadership, job content, job satisfaction, organisational commitment and general health well-being. One of the most important findings of the model is that transformational leadership behaviours can influence the quality of the working life of nurses through indirect pathways. Nurse leaders play a challenging role in the workplace and contribute to the effectiveness of a health care organisation. The present study provides clear information about the contribution of nursing transformational leadership behaviours in hospital settings. A model was proposed to examine the relationships between nursing transformational leadership and the mental health outcomes of nurses. The result showed high statistically significant correlations between job satisfaction and organisational commitment. Both job satisfaction and organisational commitment are strong predictors of nurses’ performance [65], but how does leader behaviour influence staff satisfaction? Supervisor support is one of the factors which influence psychosocial work characteristics. The present study shows a statistically significant correlation between transformational leadership behaviours and supervisor support (*r* = .735). In the proposed model, transformational leadership behaviours correlated positively with supervisor support (β = .76). Nursing staff who were more satisfied with their work had a better quality of working life. When nurses were satisfied with their employment, patient satisfaction increased [66]. As employees spend around half of their waking life at work, the workplace should be the best area to improve employees’ health behaviours. The results of the present study suggest that encouraging leaders to use transformational leadership behaviours may be helpful in enhancing organisational commitment. Thus, transformational leadership style can be a health promotion intervention applied within a health care setting.
